# A Suzuki-type multivalued contraction on weak partial metric spaces and applications

**DOI:** 10.1186/s13660-018-1866-9

**Published:** 2018-10-05

**Authors:** Hassen Aydi, M. A. Barakat, Zoran D. Mitrović, Vesna Šešum-Čavić

**Affiliations:** 1 Department of Mathematics, College of Education in Jubail, Imam Abdulrahman Bin Faisal University, Jubail, Saudi Arabia; 2 Department of Medical Research, China Medical University Hospital, China Medical University, Taichung, Taiwan; 3 Department of Mathematics, Faculty of Sciences, Al-Azhar University, Assiut, Egypt; 4 Nonlinear Analysis Research Group, Ton Duc Thang University, Ho Chi Minh City, Vietnam; 5 Faculty of Mathematics and Statistics, Ton Duc Thang University, Ho Chi Minh City, Vietnam; 6 Vienna University of Technology, Institute of Computer Languages, Vienna, Austria

**Keywords:** 47H10, 54H25, 05C40, Weak partial metric, $\mathcal{H}^{+}$-type Pompeiu–Hausdorff metric, Suzuki-type fixed point result

## Abstract

Based on a recent paper of Beg and Pathak (Vietnam J. Math. 46(3):693–706, 2018), we introduce the concept of $\mathcal{H}_{q}^{+}$-type Suzuki multivalued contraction mappings. We establish a fixed point theorem for this type of mappings in the setting of complete weak partial metric spaces. We also present an illustrated example. Moreover, we provide applications to a homotopy result and to an integral inclusion of Fredholm type. Finally, we suggest open problems for the class of 0-complete weak partial metric spaces, which is more general than complete weak partial metric spaces.

## Introduction

Throughout this paper, we use following notation: $\mathbb{N}$ is the set of all natural numbers, $\mathbb{R}$ is the set of all real numbers, and $\mathbb{R}^{+}$ is the set of all nonnegative real numbers.

### Definition 1.1

([2])

A partial metric on a nonempty set *X* is a function $p:X\times X\to \mathbb{R}^{+}$ such that, for all $x,y,z\in X$:

(P_1_): $x=y$ if and only if $p(x,x)=p(x,y)=p(y,y)$;
(P_2_): $p(x,x)\leq p(x,y)$;
(P_3_): $p(x,y)=p(y,x)$;
(P_4_): $p(x,y)\leq p(x,z)+p(z,y)-p(z,z)$.

The pair $(X,p)$ is called a partial metric space. Many fixed point results in partial metric spaces have been proved; see [3–17]. Recently, Beg and Pathak [1] introduced a weaker form of partial metrics called a weak partial metric.

### Definition 1.2

([1])

Let *X* be a nonempty set. A function $q:X\times X\rightarrow \mathbb{R}^{+}$ is called a weak partial metric on *X* if for all $x,y,z\in X$, the following conditions hold:

$(WP1)$
$q(x,x)=q(x,y)$ if and only if $x=y$;
$(WP2)$
$q(x, x) \le q(x, y)$;
$(WP3)$
$q(x, y) = q(y, x)$;
$(WP4)$
$q(x,y)\leq q(x,z)+q(z,y)$.

The pair $(X, q)$ is called a weak partial metric space.

Examples of weak partial metric spaces [1] are:

1. $(\mathbb{R}^{+},q)$, where $q:\mathbb{R}^{+}\times \mathbb{R} ^{+}\rightarrow \mathbb{R}^{+}$ is defined as $q(x,y)=\vert x-y \vert +1$ for $x,y\in \mathbb{R}^{+}$.
2. $(\mathbb{R}^{+},q)$, where $q:\mathbb{R}^{+}\times \mathbb{R}^{+}\rightarrow \mathbb{R}^{+}$ is defined as $q(x,y)=\frac{1}{4}\vert x-y \vert +\max \{x,y\}$ for $x,y\in \mathbb{R}^{+}$.
3. $(\mathbb{R}^{+},q)$, where $q:\mathbb{R}^{+}\times \mathbb{R}^{+}\rightarrow \mathbb{R}^{+}$ is defined as $q(x,y)=\max \{x,y\}+e^{\vert x-y \vert }+1$ for $x,y\in \mathbb{R} ^{+}$.

Notice that

- If $q(x,y)=0$, then $(WP1)$ and $(WP2)$ imply that $x=y$, but the converse need not be true.
- $(P1)$ implies $(WP1)$, but the converse need not be true.
- $(P4)$ implies $(WP4)$, but the converse need not be true.

### Example 1.1

([1])

If $X=\{[a,b]:a,b\in \mathbb{R},a\leq b\}$, then $q([a,b],[c,d])=\max \{b,d\}-\min \{a,c\}$ is a weak partial metric.

Each weak partial metric *q* on *X* generates a $T_{0}$ topology $\tau _{q}$ on *X*. Topology $\tau _{q}$ has as a base the family of open *q*-balls $\{B_{q}(x,\epsilon ):x\in X,\epsilon >0\}$, where $B_{q}(x,\epsilon )=\{y\in X:q(x,y)< q(x,x)+\epsilon \}$ for all $x\in X$ and $\epsilon >0$.

If *q* is a weak partial metric on *X*, then the function $q^{s}:X\times X\to [0,\infty )$ given by $q^{s}(x,y)=q(x,y)-\frac{1}{2}[q(x,x)+q(y,y)]$ defines a metric on *X*.

### Definition 1.3

Let $(X,q)$ be a weak partial metric space.

(i) A sequence $\{x_{n}\}$ in $(X, q)$ converges to a point $x\in X$, with respect to $\tau _{q}$ if $q(x,x)=\lim_{n\rightarrow \infty }q(x,x_{n})$;
(ii) A sequence $\{x_{n}\}$ in *X* is said to be a Cauchy sequence if $\lim_{n, m\rightarrow \infty } q(x_{n}, x_{m})$ exists and is finite;
(iii) $(X, q)$ is called complete if every Cauchy sequence $\{x_{n}\}$ in *X* converges to $x\in X$ with respect to topology $\tau _{q}$.

Clearly, we also have the following:

### Lemma 1.1

*Let* $(X,q)$ *be a weak partial metric space*. *Then*

- *A sequence* $\{x_{n}\}$ *in* *X* *is Cauchy sequence in* $(X,q)$ *if and only if it is a Cauchy sequence in the metric space* $(X, q^{s})$;
- $(X,q)$ *is complete if and only if the metric space* $(X,q^{s})$ *is complete*. *Furthermore*, *a sequence* $\{x_{n}\} $ *converges in* $(X,q^{s})$ *to a point* $x\in X$ *if and only if*
  1.1 $$ \lim_{ n, m \to \infty } q(x_{n}, x_{m}) = \lim_{n \to \infty } q(x_{n}, x) = q(x, x). $$

Let $(X,q)$ be a weak partial metric space. Let $CB^{q}(X)$ be the family of all nonempty closed bounded subsets of $(X,q)$. Here, the boundedness is given as follows: *E* is a bounded subset in $(X,q)$ if there exist $x_{0}\in X$ and $M\geq 0$ such that, for all $a\in E$, we have $a\in B_{q}(x_{0},M)$, that is, $q(x_{0},a)< q(a,a)+M$.

For $E,F\in \mathit{CB}^{q}(X)$ and $x\in X$, define

$$ q(x,E)=\inf \bigl\{ q(x,a),a\in E \bigr\} ,\qquad \delta _{q}(E,F)=\sup \bigl\{ q(a,F):a\in E \bigr\} $$

and

$$ \delta _{q}(F,E)=\sup \bigl\{ q(b,E):b\in F \bigr\} . $$

Now, $q(x,E)=0$ implies $q^{s}(x,E)=0$, where $q^{s}(x,E)=\inf \{q^{s}(x,a),a\in E\}$.

### Remark 1.1

([1])

Let $(X,q)$ be a weak partial metric space, and let *E* be a nonempty set in ($X,q$). Then

1.2 $$ a\in \overline{E} \quad \mbox{if and only if}\quad q(a,E)=q(a,a), $$

where *E̅* denotes the closure of *E* with respect to the weak partial metric *q*.

Note that *E* is closed in $(X,q)$ if and only if $E=\overline{E}$.

First, we study properties of the mapping $\delta _{q}:\mathit{CB}^{q}(X)\times \mathit{CB}^{q}(X)\rightarrow[ 0,\infty )$.

### Proposition 1.1

([1])

*Let* ($X, q$) *be a weak partial metric space*,*We have the following*:

(i) $\delta _{q}(E,E)=\sup \{q(a,a):a\in E\}$;
(ii) $\delta _{q}(E,E)\leq \delta _{q}(E,F)$;
(iii) $\delta _{q}(E,F)=0$ *implies* $E\subseteq F$;
(iv) $\delta _{q}(E,F)\leq \delta _{q}(E,H)+\delta _{q}(H,F)$ *for all* $E ,F, H\in \mathit{CB}^{q}(X)$.

### Definition 1.4

([1])

Let $(X,q)$ be a weak partial metric space. For $E,F\in \mathit{CB}^{q}(X)$, define

1.3 $$ \mathcal{H}_{q}^{+}(E,F)=\frac{1}{2} \bigl\{ \delta _{q}(E,F)+\delta _{q}(F,E) \bigr\} . $$

The following proposition is a consequence of Proposition 1.1.

### Proposition 1.2

([1])

*Let* $(X, q)$ *be a weak partial metric space*. *Then*, *for all* $E, F, H\in \mathit{CB}^{q}(X)$, *we have*

1. $\mathcal{H}_{q}^{+}(E,E)\leq \mathcal{H}_{q}^{+}(E,F)$;
2. $\mathcal{H}_{q}^{+}(E,F)=\mathcal{H}_{q}^{+}(F,E)$;
3. $\mathcal{H}_{q}^{+}(E,F)\leq \mathcal{H}_{q}^{+}(E,H)+\mathcal{H}_{q}^{+}(H,F)$.

The mapping $\mathcal{H}_{q}^{+}:\mathit{CB}^{q}(X)\times \mathit{CB}^{q}(X)\rightarrow[ 0,+\infty )$, is called the $\mathcal{H}^{+}$-type Pompeiu–Hausdorff metric induced by *q*.

### Definition 1.5

([1])

Let $(X,q)$ be a complete weak partial metric space. A multivalued map $T:X\rightarrow \mathit{CB}^{q}(X)$ is called an $\mathcal{H}_{q}^{+}$-contraction if

$(1^{\circ })$
there exists *k* in $(0, 1)$ such that
  1.4 $$ \mathcal{H}_{q}^{+} \bigl(Tx\setminus \{x\}, Ty\setminus \{y \} \bigr) \le k q(x, y) \quad \mbox{for every } x, y \in X, $$
$(2^{\circ })$
for all *x* in $X, y$ in *Tx*, and $\epsilon > 0$, there exists *z* in *Ty* such that
  1.5 $$ q(y, z) \leq \mathcal{H}_{q}^{+}(Ty, Tx) + \epsilon . $$

Beg and Pathak [1] proved the following fixed point theorem.

### Theorem 1.1

([1])

*Let* $(X,q)$ *be a complete weak partial metric space*. *Every* $\mathcal{H}_{q}^{+}$-*type multivalued contraction mapping* $T:X\rightarrow \mathit{CB}^{q}(X)$ *with Lipschitz constant* $k<1$ *has a fixed point*.

In this paper, we generalize the concept of $\mathcal{H}_{q}^{+}$-type multivalued contractions by introducing $\mathcal{H}_{q}^{+}$-type Suzuki mult-valued contraction mappings.

## Fixed point results

First, let $\psi : [0, 1)\rightarrow (0, 1]$ be the nonincreasing function

2.1 $$ \psi (r)= \textstyle\begin{cases} 1& \mbox{if } 0\leq r < \frac{1}{2}, \\ 1-r& \mbox{if } \frac{1}{2}\leq r < 1. \end{cases} $$

Now, we state a fixed point result for $\mathcal{H}_{q}^{+}$-type Suzuki multivalued contraction mappings.

### Theorem 2.1

*Let* $(X,q)$ *be a complete weak partial metric space*, *and let* $F : X \to \mathcal{CB}^{q}(X)$ *be a multivalued mapping*. *Let* $\psi : [0, 1)\rightarrow (0, 1]$ *be the nonincreasing function defined by* (2.1). *Suppose that there exists* $0 \leq s < 1$ *such that* *T* *satisfies the condition*

2.2 $$ \psi (s)q(x,Fx)\leq q(x,y) \quad \textit{implies } \mathcal{H}_{q}^{+} \bigl(Fx\setminus \{x\},Fy\setminus \{y\} \bigr)\leq s q(x,y) $$

*for all* $x,y \in X$. *Suppose also that*, *for all* *x* *in* $X, y$ *in* *Fx*, *and* $t > 1$, *there exists* *z* *in* *Fy* *such that*

2.3 $$ q(y, z) \le t \mathcal{H}_{q}^{+}(Fy, Fx). $$

*Then* *F* *has a fixed point*.

### Proof

Let $s_{1}\in (0, 1)$ be such that $0\leq s \leq s_{1} < 1$ and $w_{0}\in X$. Since $Fw_{0}$ is nonempty, it follows that if $w_{0} \in Fw_{0}$, then the proof is completed. Let $w_{0}\notin Fw_{0}$. Then there exists $w_{1}\in Fw_{0}$ such that $w_{1}\neq w_{0}$.

Similarly, there exists $w_{2}\in Fw_{1}$ such that $w_{1}\neq w_{2}$, and from (2.3) we have

2.4 $$ q(w_{1},w_{2})\leq \frac{1}{\sqrt{s_{1}}}H^{+}_{q}(Fw_{0},Fw_{1}). $$

Since

$$ \psi (s)q(w_{1},Fw_{1})\leq q(w_{1},Fw_{1}) \leq q(w_{1},w_{2}), $$

from (2.2) and (2.4) we get

$$\begin{aligned} q(w_{1},w_{2})&\leq \frac{1}{\sqrt{s_{1}}}H^{+}_{q}(Fw_{0},Fw_{1})\leq \frac{1}{\sqrt{s_{1}}}H^{+}_{q} \bigl(Fw_{0} \setminus \{w_{0}\},Fw_{1}\setminus \{w_{1}\} \bigr) \\ & \leq \frac{1}{\sqrt{s_{1}}}. s .q(w_{0},w_{1})< \sqrt{s_{1}}. q(w_{0},w_{1}). \end{aligned}$$

By repeating this process *n* times we obtain

2.5 $$ q(w_{n},w_{n+1})\leq (\sqrt{s_{1}})^{n} \cdot q(w_{0},w_{1}). $$

Hence

2.6 $$ \lim_{n\to \infty }q(w_{n},w_{n+1})=0. $$

Now we prove that $\{w_{n}\}$ is a Cauchy sequence in $(X,q^{s})$. For all $m\in N$, we have

$$\begin{aligned} q^{s}(w_{n},w_{n+m})&= q(w_{n},w_{n+m})- \frac{1}{2} \bigl[q(w_{n},w_{n})+q(w_{n+m},w_{n+m}) \bigr] \\ &\leq q(w_{n},w_{n+m}) \\ &\leq q(w_{n},w_{n+1})+q(w_{n+1},w_{n+2})+ \cdots+q(w_{n+m-1},w_{n+m}) \\ & \leq \bigl[(\sqrt{s_{1}})^{n}+(\sqrt{s_{1}})^{n+1}+ \cdots+(\sqrt{s_{1}})^{n+m-1} \bigr]q(w_{0},w_{1}) \\ &\leq (\sqrt{s_{1}})^{n}\frac{1}{1-\sqrt{s_{1}}}q(w_{0},w_{1}). \end{aligned}$$

Hence

2.7 $$ \lim_{n\to \infty }q^{s}(w_{n},w_{n+m})=0. $$

This implies that $\{w_{n}\}$ is a Cauchy sequence in the complete metric space $(X,q^{s})$. It follows that there exists $u\in X $ such that

2.8 $$ \lim_{n\to \infty }q(w_{n},u)=\lim _{n,m\to \infty }q(w_{n},w_{m})=q(u,u). $$

From $(WP2)$ we obtain

2.9 $$ \frac{1}{2} \bigl[q(w_{n},w_{n})+q(w_{n+1},w_{n+1}) \bigr]\leq q(w_{n},w_{n+1}). $$

By taking the limit as $n\to \infty $ from (2.6) we get

2.10 $$ \lim_{n\to \infty }q(w_{n},w_{n})= \lim_{n\to \infty }q(w_{n+1},w_{n+1})=\lim _{n\to \infty }q(w_{n},w_{n+1})=0. $$

Also, from (2.7) and (2.10) we find

2.11 $$ \lim_{n\to \infty }q^{s}(w_{n},w_{n+m})=0= \lim_{n\to \infty }q(w_{n},w_{n+m})-\frac{1}{2} \lim_{n\to \infty } \bigl[q(w_{n},w_{n})+q(w_{n+m},w_{n+m}) \bigr]. $$

Therefore

2.12 $$ \lim_{n\to \infty }q(w_{n},w_{n+m})=0= \lim_{n\to\infty }q(w_{n},u)=q(u,u). $$

Now, we prove that

2.13 $$ q(u,Fx)\leq 2s q(u,x)\quad \mbox{for all } x\in X\setminus \{u\}. $$

Since $\lim_{n\to \infty } q(w_{n},u)=0$, there exists $n_{0}\in \mathbb{N}$ such that

$$ q(w_{n},u)\leq \frac{1}{3}q(x,u)\quad \mbox{for all } n\geq n_{0}. $$

Then

$$\begin{aligned} \psi (s)q(w_{n},Fw_{n}) &\leq q(w_{n},Fw_{n}) \\ &\leq q(w_{n},w_{n+1}) \\ &\leq q(w_{n},u)+q(u,w_{n+1}) \\ &\leq \frac{1}{3}q(u,x)+ \frac{1}{3}q(u,x) \\ &\leq q(u,x)-\frac{1}{3}q(u,x) \\ &\leq q(u,x)-q(u,w_{n})\leq q(x,w_{n}). \end{aligned}$$

This implies that

$$ H^{+}_{q}(Fw_{n},Fx)\leq s q(w_{n},x). $$

Since $w_{n+1}\in Fw_{n}$, we have

$$\begin{aligned} q(w_{n+1},Fx)&\leq \delta _{q}(Fw_{n},Fx) \\ &\leq 2H^{+}_{q}(Fw_{n},Fx) \\ &\leq 2 s q(w_{n},x) \\ &\leq 2s \bigl[q(w_{n},u)+q(u,x) \bigr]. \end{aligned}$$

By taking the limit as $n\to \infty $ we get

2.14 $$ \lim_{n\to \infty }q(w_{n+1},Fx)\leq 2s q(u,x). $$

Also, since

$$ q(u,Fx)\leq q(u,w_{n+1})+q(w_{n+1},Fx) $$

and

$$ q(w_{n+1},Fx)\leq q(w_{n+1},w_{n})+q(w_{n},u)+q(u,Fx), $$

we have

2.15 $$ \lim_{n\to \infty }q(w_{n+1},Fx)= q(u,Fx). $$

From (2.14) and (2.15) we find that

2.16 $$ q(u,Fx)\leq 2s q(u,x)\quad \mbox{for all } x\in X\setminus \{u \}. $$

We claim that

$$ H^{+}_{q}(Fx,Fu)\leq s q(u,u) \quad \mbox{for all } x\in X. $$

If $x=u$, then at that point, this clearly holds. So, let $x\neq u$. Then for every positive integer $n\in \mathbb{N}$, there exists $y_{n} \in Fx $ such that

$$ q(u,y_{n})\leq q(u,Fx)+\frac{1}{n} q(u,x). $$

Therefore

2.17 $$\begin{aligned} q(x,Fx)&\leq q(x,y_{n}) \\ &\leq q(x,u)+q(u,y_{n}) \\ &\leq q(x,u)+q(u,Fx)+\frac{1}{n} q(x,u). \end{aligned}$$

From (2.16) and (2.17) we get

2.18 $$\begin{aligned} q(x,Fx)&\leq q(u,x)+2s q(u,x)+\frac{1}{n} q(x,u) \end{aligned}$$

2.19 $$\begin{aligned} &= \biggl[1+2s+\frac{1}{n} \biggr]q(x,u). \end{aligned}$$

Hence

$$\begin{aligned} \frac{1}{1+2s+\frac{1}{n}} q(x,Fx)\leq q(u,x). \end{aligned}$$

This implies that

$$\begin{aligned} H^{+}_{q}(Fu,Fx)\leq s q(u,x). \end{aligned}$$

Finally, we show that $u\in Fu$. For this,

$$\begin{aligned} q(u,Fu)&=\lim_{n\to \infty }q(w_{n+1},Fu) \\ &\leq \lim _{n\to \infty }\delta _{q}(Fw_{n},Fu) \\ &\leq 2\lim_{n\to \infty }H^{+}_{q}(Fw_{n},Fu) \\ &\leq 2 s\lim_{n\to \infty } q(w_{n},u)=0. \end{aligned}$$

We deduce that $q(u,u)=q(u,Fu)=0$. Since *Fu* is closed, $u\in \overline{Fu}=Fu$. □

We provide the following example.

### Example 2.1

Let $X=\{0,\frac{1}{2},1\}$ and define a weak partial metric $q:X\times X \to [0,\infty ) $ as follows: $q(0,0)=0$, $q(\frac{1}{2}, \frac{1}{2})=\frac{1}{3}$, $q(1,1)=\frac{1}{4}$, $q(0,\frac{1}{2})=q(\frac{1}{2},0)=\frac{1}{2}$, $q(\frac{1}{2},1)=q(1,\frac{1}{2})=\frac{3}{4}$, and $q(1,0)=q(0,1)=1$. It is clear that $(X,q)$ is a weak partial metric space. Note that

$$ q(1,0)=1\nleq q \biggl(1,\frac{1}{2} \biggr)+q \biggl(\frac{1}{2},0 \biggr)-q \biggl(\frac{1}{2},\frac{1}{2} \biggr)=\frac{3}{4}+ \frac{1}{2}-\frac{1}{3}. $$

Then $(X,q)$ is not a partial metric space. Define the mapping $F:X\to \mathit{CB}^{q}(X)$ by $F(0)=F(\frac{1}{2})=\{0\}$ and $F(1)=\{0,\frac{1}{3}\}$. Choose $s=0.5$. From the definition of *ψ* we have $\psi (s)=1$.

To prove the contraction condition (2.2), we need the following cases:

Case 1. At $x=0$, we have

$$ \psi (s)q \bigl(0,F(0) \bigr)=q(0,0)=0\leq q(0,y) \quad \mbox{for all } x\in X. $$

For $y=0$, we have

$$ H^{+}_{q} \bigl(F(0)\setminus \{0\},F(0)\setminus \{0\}\bigr)=H^{+}_{q}(\phi ,\phi )=0\leq s q(0,0). $$

For $y=\frac{1}{2}$, we get

$$ H^{+}_{q} \biggl(F(0)\setminus \{0\},F \biggl(\frac{1}{2} \Bigm\backslash \biggl\{ \frac{1}{2} \biggr\} \biggr)=H^{+}_{q} \bigl(\phi ,\{0\} \bigr)=0\leq s q \biggl(0, \frac{1}{2} \biggr). $$

If f $y=1$, then

$$ H^{+}_{q} \bigl(F(0)\setminus \{0\},F(1)\setminus \{1\} \bigr)=H^{+}_{q} \biggl(\phi , \biggl\{ 0,\frac{1}{2} \biggr\} \biggr)=0\leq s q(0,1). $$

Case 2. At $x=\frac{1}{2}$, we have

$$ \psi (s)q \biggl(\frac{1}{2},F \biggl(\frac{1}{2} \biggr) \biggr)=q \biggl(\frac{1}{2},0 \biggr)=\frac{1}{2}\leq q \biggl( \frac{1}{2},y \biggr)\quad\mbox{for all } y\in X\Bigm\backslash \biggl\{ \frac{1}{2} \biggr\} . $$

Similarly, if $y=0, then$

$$ H^{+}_{q} \biggl(F \biggl(\frac{1}{2} \Bigm\backslash \biggl\{ \frac{1}{2} \biggr\} ,F(0)\setminus \{0\} \biggr)=H^{+}_{q} \bigl(\{0\},\phi \bigr)=0\leq s q \biggl(\frac{1}{2},0 \biggr), $$

If $y=1$, then

$$ H^{+}_{q} \biggl(F \biggl(\frac{1}{2} \biggr)\Bigm\backslash \biggl\{ \frac{1}{2} \biggr\} ,F(1)\setminus \{1\} \biggr)=H^{+}_{q}\biggl(\{0\}, \biggl\{ 0,\frac{1}{2} \biggr\} \biggr)=\frac{1}{4}< s q \biggl(\frac{1}{2},1 \biggr)=\frac{3}{8}. $$

Case 3. At $x=1$, we have

$$ \psi (s)q \bigl(1,F(1) \bigr)=q \biggl(1,\frac{1}{2} \biggr)= \frac{3}{4} \leq q(1,y)\quad\mbox{for all } y\in X\setminus \{1\}. $$

Again, if $y=0$, then

$$ H^{+}_{q} \bigl(F(1)\setminus \{1\},F(0)\setminus \{0\} \bigr)=H^{+}_{q} \biggl( \biggl\{ 0,\frac{1}{2} \biggr\} , \phi \biggr)=0\leq s q(1,0). $$

If $y=\frac{1}{2}$, then

$$ H^{+}_{q} \biggl(F(1)\setminus \{1\},F \biggl( \frac{1}{2} \Bigm\backslash \biggl\{ \frac{1}{2} \biggr\} \biggr)=H^{+}_{q} \biggl( \biggl\{ 0,\frac{1}{2} \biggr\} ,\{0\} \biggr)=\frac{1}{4}< s q \biggl(1,\frac{1}{2} \biggr)= \frac{3}{8}. $$

Finally, we will enquire the condition (2.3) with $t=2$. For this, we discuss the following situations:

(i) If $x=0$ or $x=\frac{1}{2}$, then $y\in F(0)=F(\frac{1}{2})=\{0\}$. This yields that $y=0$, so there exists $z\in F(y)$ such that
  $$ 0=q(y,z)\leq 2 H^{+}_{q} \bigl(F(x),F(y) \bigr). $$
(ii) If $x=1$, then $y\in F(1)=\{0,\frac{1}{2}\}$. If $y=0$, then $z=0$, and condition (2.3) is satisfied.
  Also, If $y=\frac{1}{2}$, then $z=0$, so that
  $$ \frac{1}{2}=q(y,z)= 2 H^{+}_{q} \biggl(F(1),F \biggl( \frac{1}{2} \biggr) \biggr)=\frac{1}{2}. $$
  Therefore all conditions of Theorem 2.1 are satisfied, and the function *F* has a fixed point $u=0$.

On the other hand, the result of Beg and Pathak [1] is not applicable. Indeed,

$$ H^{+}_{q} \bigl(F(1)\setminus \{1\},F(1)\setminus \{1\} \bigr)= \frac{1}{3}>\frac{1}{2} q(1,1)=\frac{1}{8}. $$

## Applications

First, we present an application concerning a homotopy result for complete weak partial metric spaces.

### Theorem 3.1

*Let* $(X, q)$ *be a complete weak partial metric space*, *let* *D* *be an open subset of* *X*, *and let* *W* *be a closed subset of* *X* *with* $D\subset W$. *Let* $F : W \times [0, 1] \to \mathit{CB}^{q} (X)$ *be an operator satisfying*:

(i) $x\notin F (x, t)$ *for each* $x \in W \setminus D$ *and each* $t\in [0, 1]$;
(ii) *there exists* $s \in (0, \frac{1}{2})$ *such that*, *for each* $t \in [0, 1]$ *and each* $x, y \in W$, *we have*
  $$ \psi (s) q \bigl(x,F(x,t) \bigr)\leq q(x,y)\Rightarrow H^{+}_{q} \bigl( F (x, t) \setminus \{x\}, F (y, t) \setminus \{y\} \bigr) \leq s q(x, y); $$
(iii) *for all* $x \in W$, $y \in F (x, t)$, *and* $h > 1$, *there exists* $z \in F (y, t)$ *such that*
  $$ q(y, z) \leq h H^{+}_{q} \bigl(F (y, t), F (x, t) \bigr); $$
(iv) *there exists a continuous function* $\eta : [0, 1] \to \mathbb{R}$ *such that*
  $$ H^{+}_{q} \bigl( F (x, t_{1}) \setminus \{x\}, F (x, t_{2}) \setminus \{x\} \bigr) \leq s\bigl\vert \eta (t_{1}) -\eta (t_{2}) \bigr\vert $$
  *for all* $t_{1}, t_{2} \in [0, 1]$ *and* $x \in W$;
(v) *if* $x \in F (x, t)$, *then* $F (x, t) = \{x\}$. *Then* $F (\cdot , 0)$ *has a fixed point if and only if* $F (\cdot , 1)$ *has a fixed point*.

### Proof

Define the set

$$ \Delta := \bigl\{ t\in [0,1] ;x\in F(x, t) \mbox{ for some } x\in D \bigr\} . $$

Since $F (\cdot , 0)$ has a fixed point, from condition (i), we get $0\in \Delta $, so $\Delta \neq\phi $. First, we want to show that Δ is an open set. Let $t_{1}\in \Delta $ and $x_{1}\in D$ be such that $x_{1}\in F(x_{1},t_{1} )$. Since *D* is open in $(X,q)$, there exists $r>0$ such that $B(x_{1},r)\subset D$. Consider $\epsilon =(\frac{1-2s}{2})(q(x_{1},x_{1})+r)>0$. Since *η* is continuous at $t_{1}$, there exists $\delta (\epsilon )>0$ such that $\vert \eta (t)-\eta (t_{1}) \vert < \epsilon $ for all $t\in (t_{1}-\delta (\epsilon ),t_{1}+\delta (\epsilon ))$.

Let $t\in (t_{1}-\delta (\epsilon ),t_{1}+\delta (\epsilon ))$ and $x\in B(x_{1},r)=\{x\in X ;q(x_{1},x)\leq q(x_{1},x_{1})+r\}$. Since $x_{1}\in F(x_{1},t_{1} )$, from $(WP2)$ we have

$$ \psi (s)q \bigl(x_{1},F(x_{1},t_{1} ) \bigr) \leq q(x_{1},x_{1})\leq q(x_{1},x)\quad\mbox{for all } x\in X. $$

Thus

$$\begin{aligned} q \bigl(x_{1},F(x,t) \bigr)&\leq 2H^{+}_{q} \bigl(F(x,t),F(x_{1},t_{1}) \bigr) \\ &\leq 2 \bigl[H^{+}_{q} \bigl(F(x,t),F(x,t_{1}) \bigr)+H^{+}_{q} \bigl(F(x,t_{1}),F(x_{1},t_{1}) \bigr) \bigr] \\ &=2 \bigl[H^{+}_{q} \bigl(F(x,t)\setminus \{x \},F(x,t_{1})\setminus \{x\} \bigr) +H^{+}_{q} \bigl(F(x,t_{1})\setminus \{x \},F(x_{1},t_{1})\setminus \{x_{1}\} \bigr) \bigr] \\ &\leq 2 \bigl[\bigl\vert \eta (t) - \eta (t_{1}) \bigr\vert +s q(x,x_{1}) \bigr] \\ &\leq 2 \bigl[\epsilon +s \bigl(q(x_{1},x_{1})+r \bigr) \bigr] \\ &\leq 2 \biggl[ \biggl(\frac{1-2s}{2} \biggr) \bigl(q(x_{1},x_{1})+r \bigr)+s \bigl(q(x_{1},x_{1})+r \bigr) \biggr] \\ &\leq q(x_{1},x_{1})+r. \end{aligned}$$

Therefore $F(x,t)\subset B(x_{1},r)$. Since $F(\cdot ,t):B(x_{1},r)\to \mathit{CB}^{q}(X)$ for each fixed $t\in (t_{1}-\delta (\epsilon ),t-1+\delta (\epsilon ))$ and (ii) holds, all the hypotheses of Theorem 2.1 are satisfied. We conclude that $F(\cdot ,t)$ has a fixed point in $B(x_{1},r)\subset W$. This fixed point must be in *D* due to (i). Hence $(t_{1}-\delta (\epsilon ),t-1+\delta (\epsilon ))\subset \Delta $, and therefore Δ is open in $[0,1]$.

Second, we prove that Δ is closed in $[0,1]$. To show this, choose a sequence $\{t_{n}\}$ in Δ such that $t_{n}\to t^{*}\in [0,1]$ as $n\to \infty $. We must show that $t^{*} \in \Delta $. By the definition of Δ there exists $x_{n} \in D$ with $x_{n} \in F(x_{n},t_{n})$. Then

$$ \psi (s)q \bigl(x_{n},F(x_{n},t_{n} ) \bigr)\leq q(x_{n},x_{n})\leq q(x_{n},x)\quad\mbox{for all } x \in X. $$

This implies that, for all positive integers $m, n\in \mathbb{N}$, using (v) and $(Wh3)$, we have

$$\begin{aligned} q(x_{n},x_{m}) &\leq 2H^{+}_{q} \bigl(F(x_{n},t_{n}), F (x_{m},t_{m}) \bigr) \\ & \leq 2H^{+}_{q} \bigl(F(x_{n},t_{n}), F (x_{n},t_{m}) \bigr)+2H^{+}_{q} \bigl(F (x_{n},t_{m}), F (x_{m},t_{m}) \bigr) \\ & =2H^{+}_{q} \bigl(F(x_{n},t_{n}) \setminus \{x_{n}\},F(x_{n},t_{m})\setminus \{x_{n}\} \bigr) \\ &\quad {} +2H^{+}_{q} \bigl(F(x_{n},t_{m}) \setminus \{x_{n}\},F(x_{m},t_{m})\setminus \{x_{m}\} \bigr) \\ & \leq 2s\bigl\vert \eta (t_{n})-\eta (t_{m}) \bigr\vert +2s q(x_{n},x_{m}). \end{aligned}$$

This implies that

$$ q(x_{n},x_{m})\leq \frac{2s}{1-2s} \bigl(\bigl\vert \eta(t_{n})-\eta (t_{m}) \bigr\vert \bigr). $$

Hence $\lim_{n,m\to \infty }q(x_{n},x_{m})=0$. Therefore $\{x_{n}\}$ is a Cauchy sequence in $(X, q)$. Since $(X, q)$ is complete, there exists $x^{*}\in W$ such that

$$ q \bigl(x^{*} ,x^{*} \bigr) = \lim_{n\to \infty }q \bigl(x^{*},x_{n} \bigr)=\lim_{n,m\to \infty } q(x_{n},x_{m})=0. $$

On the other hand, we have

$$\begin{aligned} q \bigl(x_{n},F \bigl(x^{*} ,t^{*} \bigr) \bigr)& \leq 2H^{+}_{q} \bigl(F (x_{n},t_{n}), F \bigl(x^{*} ,t^{*} \bigr) \bigr) \\ & \leq 2H^{+}_{q} \bigl(F (x_{n},t_{n}), F \bigl(x_{n},t^{*} \bigr)+2H^{+}_{q} \bigl(F \bigl(x_{n},t^{*} \bigr) \bigr), F \bigl(x^{*} ,t^{*} \bigr) \bigr) \\ &=2 H^{+}_{q} \bigl( F(x_{n},t_{n}) \setminus \{x_{n}\},F \bigl(x_{n},t^{*} \bigr) \setminus \{x_{n}\} \bigr) \\ &\quad {} +2H^{+}_{q} \bigl( F \bigl(x_{n},t^{*} \bigr)\setminus \{x_{n}\},F \bigl(x^{*} ,t^{*} \bigr)\setminus \bigl\{ x^{*} \bigr\} \bigr) \\ & \leq 2s\bigl\vert \eta (t_{n})-\eta \bigl(t^{*} \bigr) \bigr\vert +2sq \bigl(x_{n},x^{*} \bigr). \end{aligned}$$

Taking the limit as $n\to \infty $ in the above inequality, we get

$$ q \bigl(x^{*} ,F \bigl(x^{*} ,t^{*} \bigr) \bigr)= \lim_{n\to \infty } q \bigl(x_{n},F \bigl(x^{*} ,t^{*} \bigr) \bigr)=0. $$

It follows that $x^{*}\in F(x^{*} ,t^{*} )$. Thus $t^{*}\in \Delta $, and hence Δ is closed in $[0,1]$. By the connectedness of $[0,1]$ we have $\Delta =[0,1]$.

The reverse implication easily follows by applying the same strategy. This completes the proof. □

Now, we give another application to the solvability of integral inclusions of Fredholm type. Let $I=[0,1]$, and let $C(I,\mathbb{R})$ be the space of all continuous functions $f :I\to R$. Consider the weak partial metric on *X* given by

$$ q(x,y)=\sup_{t\in I} \bigl\vert x(t)-y(t) \bigr\vert +\alpha $$

for all $x,y\in C(I,R)$ and $\alpha >0$. We have $q^{s}(x,y)=\sup_{t\in I} \vert x(t)-y(t) \vert $, so by Lemma 1.1 $(C(I,\mathbb{R}),q)$ is a complete weak partial metric space. Denote by $P_{cv}(\mathbb{R})$ the family of all nonempty compact and convex subsets of $\mathbb{R}$ and by $P_{cl}(\mathbb{R})$ the family of all nonempty closed subsets of $\mathbb{R}$.

### Theorem 3.2

*Consider the integral inclusion of Fredholm type*

3.1 $$ h(t)\in f(t)+ \int ^{1}_{0} K \bigl(t,u,h(u) \bigr)\,du, \quad t\in [0,1]. $$

*Suppose that*:

(i) $K:I\times I\times R\to P_{cv}(\mathbb{R})$ *is such that* $K_{h}(t,u):=K(t,u,h(u))$ *is a lower semicontinuous for all* $(t,u)\in I\times I$ *and* $h\in C(I,\mathbb{R})$,
(ii) $f \in C(I,R)$;
(iii) *for each* $t\in I$, *there exists* $l(t,\cdot )\in L^{1}(I)$ *such that* $\sup_{ t\in I} \int ^{1}_{0} l(t,u)\,du = \frac{s}{2}$ *with* $s\in [0,1)$ *and*
  $$ H^{+}_{q} \bigl( K \bigl(t,u,h(u) \bigr),K \bigl(t,u,r(u) \bigr) \bigr) \leq l(t,u) \Bigl(\sup_{u\in I}\bigl\vert h(u)-r(u) \bigr\vert +\alpha \Bigr) $$
  *for all* $t,u\in I$ *and all* $h,r\in C(I,\mathbb{R})$.

*Then the integral inclusion* (3.1) *has at least one solution in* $C(I,\mathbb{R})$.

### Proof

Consider the multivalued operator $T:C(I,R)\to P_{CL}(C(I,R))$ defined by

$$ Tx(t)= \biggl\{ h\in C(I,\mathbb{R}) \mbox{ such that } h(t)\in f(t)+ \int ^{1}_{0} K \bigl(t,u,x(u) \bigr)\,du,t \in I \biggr\} $$

for $x\in C(I,\mathbb{R})$. For each $K_{x}(t,u):I\times I \to P_{cv}(\mathbb{R})$, by the Michael selection theorem there exists a continuous operator $k_{x}: I\times I\to \mathbb{R}$ such that $k_{x}(t,u)\in K_{x}(t,u)$ for all $t,u\in I$. This implies that $f(t)+ \int ^{1}_{0} k_{x}(t,u)\,du\in Tx $, and so $Tx\neq \emptyset $. It is easy to prove that *Tx* is closed, and so we omit the details (see also [18]). This implies that *Tx* is closed in $(C(I,\mathbb{R}),q)$.

Now, we will show that *T* is $H^{+}_{q}$-type Suzuki multivalued contraction mapping. Let $x_{1}, x_{2}\in C(I,\mathbb{R})$ and $h\in Tx$. Then there exists $k_{x_{1}}(t,u)\in K_{x_{1}}(t,u)$ with $t,u\in I$ such that $h(t)=f(t)+ \int ^{1}_{0} k_{x}(t,u)\,du,t\in I$. Also, by hypothesis (iii),

$$ H^{+}_{q} \bigl( K \bigl(t,u,x_{1}(u) \bigr),K \bigl(t,u,x_{2}(u) \bigr) \bigr) \leq l(t,u) \Bigl(\sup _{u\in I}\bigl\vert x_{1}(u)-x_{2}(u) \bigr\vert +\alpha \Bigr)\quad \forall t,u\in I. $$

Then there exists $z(t,u)\in K_{x_{2}}(t,u)$ such that

$$ \bigl\vert k_{x_{1}}(t,u)-z(t,u) \bigr\vert +n \leq l(t,u) \bigl[ \bigl\vert x_{1}(u)-x_{2}(u) \bigr\vert + \alpha \bigr] $$

for all $t,u\in I$. Now, we define the multivalued operator $M(t,u)$ by

$$ M(t,u)=K_{x_{2}}(t,u)\cap \bigl\{ m\in \mathbb{R}, \bigl\vert k_{x_{1}}(t,u)-m \bigr\vert + \alpha \leq l(t,u) \bigl(\bigl\vert x_{1}(u)-x_{2}(u) \bigr\vert +\alpha \bigr) \bigr\} $$

for $t,u\in I$. Since *M* is a lower semicontinuous operator, there exists a continuous operator $k_{x_{2}} : I\times I \to \mathbb{R}$ such that $k_{x_{2}}(t,u)\in M(t,u)$ for all $t,u\in I$ and

$$ w(t)=f(t)+ \int ^{1}_{0} k_{x_{2}}(t,u)\,du\in f(t)+ \int ^{1}_{0} K \bigl(t,u,x_{2}(u) \bigr)\,du. $$

Therefore

$$\begin{aligned} q \bigl(h(t),Tx_{2}(t) \bigr)&\leq q \bigl(h(t),w(t) \bigr) \\ &=\sup _{t\in I}\bigl\vert h(t)-w(t) \bigr\vert +\alpha \\ & =\sup_{t\in I}\biggl\vert \int ^{1}_{0} \bigl[k_{x_{1}}(t,u)-k_{x_{2}}(t,u) \bigr]\,du \biggr\vert +\alpha \\ & \leq \sup_{t\in I}\int ^{1}_{0} \bigl(\bigl\vert k_{x_{1}}(t,u)-k_{x_{2}}(t,u) \bigr\vert +\alpha -\alpha \bigr)\,du+\alpha \\ & \leq \sup_{t\in I}\int ^{1}_{0}l(t,u) \bigl[\bigl\vert x_{1}(u)-x_{2}(u) \bigr\vert + \alpha \bigr]\,du- \int ^{1}_{0}\alpha \,du+\alpha \\ & = \Bigl(\sup_{t\in I}\bigl\vert x_{1}(u)-x_{2}(u)\bigr\vert + \alpha \Bigr)\int ^{1}_{0}l(t,u)\,du \\ & \leq s q \bigl(x_{1}(t),x_{2}(t) \bigr). \end{aligned}$$

Since $h(t)\in Tx_{1}$ is arbitrary, we have

3.2 $$\begin{aligned} \delta _{q}(Tx_{1},Tx_{2})\leq s q(x_{1},x_{2}). \end{aligned}$$

Similarly, we can get

3.3 $$\begin{aligned} \delta _{q}(Tx_{2},Tx_{1})\leq s q(x_{1},x_{2}). \end{aligned}$$

From (3.2) and (3.3) we have

$$ H^{+}_{q}(Tx_{1},Tx_{2})= \frac{\delta _{q}(Tx_{1},Tx_{2})+\delta _{q}(Tx_{2},Tx_{1})}{2}\leq s q(x_{1},x_{2}). $$

In particular, the previous inequality holds for any $t\in I$, so that

$$ \psi (s)q(x_{1},Tx_{1})\leq q(x_{1},x_{2}). $$

Thus all conditions of Theorem 2.1 are satisfied, and hence a solution of (3.1) exists. □

## Perspectives

In 2010, Romaguera [19] introduced the notions of 0-Cauchy sequences and 0-complete partial metric spaces and proved some characterizations of partial metric spaces in terms of completeness and 0-completeness. Adapting the same concepts, we introduce the concepts of 0-Cauchy sequences and 0-complete weak partial metric spaces.

### Definition 4.1

Let $(X,q)$ be a weak partial metric space.

(i) A sequence $\{x_{n}\}$in *X* is said to be 0-Cauchy if $\displaystyle \lim_{n,m\rightarrow \infty } q(x_{n}, x_{m})=0$;
(iii) $(X,q)$ is called 0-complete if every 0-Cauchy sequence $\{x_{n}\}$ in *X* converges to $x\in X$ such that $q(x,x)=0$.

Open problems: Since 0-completeness is more general than completeness, we would like to prove

(i) Theorem 1.1 and Theorem 2.1, and
(ii) a Hardy–Rogers-type result

in the class of 0-complete weak partial metric spaces.

## References

[1] Beg I., Pathak H.K. (2018). A variant of Nadler’s theorem on weak partial metric spaces with application to a homotopy result. Vietnam J. Math..

[2] Matthews S.G. (1994). Partial metric topology. Ann. N.Y. Acad. Sci..

[3] Abdeljawad T., Aydi H., Karapınar E. (2012). Coupled fixed points for Meir–Keeler contractions in ordered partial metric spaces. Math. Probl. Eng..

[4] Altun I., Sola F., Simsek H. (2010). Generalized contractions on partial metric spaces. Topol. Appl..

[5] Aydi H., Barakat A.F.H.I.M. (2017). On *ϕ*-contraction type couplings in partial metric spaces. J. Math. Anal..

[6] Aydi H., Amor S.H., Karapınar E. (2013). Berinde-type generalized contractions on partial metric spaces. Abstr. Appl. Anal..

[7] Ćirić L., Samet B., Aydi H., Vetro C. (2011). Common fixed points of generalized contractions on partial metric spaces and an application. Appl. Math. Comput..

[8] Ćojbašić R.V., Radenović S., Chauhan S. (2014). Common fixed point of generalized weakly contractive maps in partial metric spaces. Acta Math. Sci. Ser. B Engl. Ed..

[9] Di Bari C., Milojević M., Radenović S., Vetro P. (2012). Common fixed points for self-mappings on partial metric spaces. Fixed Point Theory Appl..

[10] Haghi R.H., Rezapour S., Shahzad N. (2013). Be careful on partial metric fixed point results. Topol. Appl..

[11] Kadelburg Z., Radenovic S. (2014). Fixed points under *ψ*-*α*-*β* conditions in ordered partial metric spaces. Int. J. Anal. Appl..

[12] Kadelburg Z., Nashine H.K., Radenović S. (2013). Coupled fixed points in 0-complete ordered partial metric spaces. J. Adv. Math. Stud..

[13] Lakzian H., Aydi H., Rhoades B.E. (2013). Fixed points for $(\varphi,\psi,p)$-weakly contractive mappings in metric spaces with image-distance. Appl. Math. Comput..

[14] Radenović S. (2013). Remarks on some coupled fixed point results in partial metric spaces. Nonlinear Funct. Anal. Appl..

[15] Shukla S., Radenović S. (2013). Some common fixed point theorems for *F*-contraction type mappings in 0-complete partial metric spaces. J. Math..

[16] Shukla S., Radenović S., Vetro C. (2014). Set-valued Hardy–Rogers type contraction in 0-complete partial metric spaces. Int. J. Math. Math. Sci..

[17] Vetro F., Radenović S. (2012). Nonlinear *ψ*-quasi-contractions of Ćirić-type in partial metric spaces. Appl. Math. Comput..

[18] Sîntămărian A. (2002). Integral inclusions of Fredholm type relative to multivalued *φ*-contractions. Semin. Fixed Point Theory Cluj-Napoca.

[19] Romaguera S. (2009). A Kirk type characterization of completeness for partial metric spaces. Fixed Point Theory Appl..

[20] Aydi H., Abbas M., Vetro C. (2012). Partial Hausdorff metric and Nadler’s fixed point theorem on partial metric spaces. Topol. Appl..

[21] Aydi H., Abbas M., Vetro C. (2014). Common fixed points for multivalued generalized contractions on partial metric spaces. Rev. R. Acad. Cienc. Exactas Fís. Nat., Ser. A Mat..

